# Circannual prevalence of vitamin D insufficiency in older and minoritized ethnic adults in Northern Britain: screening outcomes from a clinical trial

**DOI:** 10.1038/s41430-026-01760-z

**Published:** 2026-05-19

**Authors:** Alice Goddard, Anthony Watson, Rowena Tilbury, Bernard M. Corfe, Andrea Fairley

**Affiliations:** 1 Human Nutrition and Exercise Research Centre and School of Biomedical, Nutrition, Sports and Exercise Sciences, Faculty of Medical Sciences, Newcastle University, Newcastle upon Tyne, UK; 2 Human Nutrition and Exercise Research Centre and Population Health Sciences Institute, Faculty of Medical Sciences, Newcastle University, Newcastle upon Tyne, UK

**Keywords:** Randomized controlled trials, Nutrition

## Abstract

**Background:**

Vitamin D is essential for bone and metabolic health. Deficiency remains a global health issue, particularly among older adults and ethnic minorities with darker skin pigmentation. Data on circannual variation these groups remain sparse.

**Subjects/methods:**

This study reports vitamin D status in older adults ( ≥ 65 years) and ethnic adults ( ≥ 18 years, Fitzpatrick classes IV–VI) in northern Britain, assessed during the screening phase of a supplementation trial. Participants were screened for inclusion between December 2024 and August 2025. Serum 25-hydroxyvitamin D (25(OH)D) was assessed in dried blood spots followed by LC-MS/MS analysis.

**Results:**

In total, 299 participants were screened. Vitamin D insufficiency or deficiency ( < 50 nmol/L) was noted in 54.8% of older adults and 72.1% of ethnic individuals. These rates did not decline during summer months.

**Conclusion:**

These findings highlight persistently high rates of vitamin D insufficiency across high-risk groups in northern Britain and underscore the inadequacy of sunlight exposure as a corrective measure.

## Introduction

Vitamin D is a fat-soluble vitamin essential for bone development and maintenance, primarily by enhancing calcium, magnesium, and phosphate absorption. It is obtained through dietary sources, mainly as cholecalciferol (vitamin-D₃) found in animal-based foods and as ergocalciferol (vitamin-D₂) derived from plant sources and UV-exposed fungi. Additionally, dermal exposure to sunlight (ultraviolet-B) enables cutaneous synthesis of vitamin D through the photoconversion of 7-dehydrocholesterol to previtamin-D3 and thermal isomerisation to form cholecalciferol.

Vitamin D is absorbed in the small intestine and transported to the liver by chylomicrons before undergoing both hepatic and renal hydroxylation, forming key metabolites, 25- hydroxyvitaminD (25(OH)D), the primary circulating form, and 1,25-dihydroxyvitaminD (1,25(OH)2D), the active hormonal form, respectively. Whilst both are necessary for biological functions, 25(OH)D, is the most widely recognised as a reliable biomarker for assessing vitamin D status in humans [1]. Sufficient levels are defined as >50 nmol/L, with insufficiency at 31–49 nmol/L and deficiency at <30 nmol/L [2]. Supplementation, achieved through specific food supplements or fortified dietary sources, is recommended to maintain adequate vitamin D levels in various high-risk populations [3].

Vitamin D deficiency is a widespread public health concern linked not only to bone and immune health, but also to increased risk of chronic diseases such as cardiovascular disease and diabetes [4, 5]. Although vitamin D deficiency is commonly viewed as a seasonal issue due to reduced sunlight exposure during winter, emerging evidence suggests that some demographic groups remain at risk year-round. Within the UK, certain subpopulations including various ethnic groups are at greater risk of vitamin D deficiency. In 2021, up to 92% of UK-dwelling South Asians (*n* = 5918), aged 40–69 years and 84% of UK-dwelling African-Caribbean individuals (*n* = 4046, aged ≥40 years) had insufficient 25(OH)D levels ( < 50 nmol/L) [6, 7] demonstrating how individuals within these communities are at greater risk of deficiency. Likewise, deficiency is widespread among older adults globally, occurring irrespective of season [8] reinforcing the need for targeted public health strategies addressing these vulnerable groups.

Our current study (ISRCTN13778806) investigates the nutrikinetics of vitamin D supplementation in older adults and ethnic individuals with darker skin pigmentation (as quantified by the Fitzpatrick classes IV, V, VI) currently residing in Britain. This report focuses on the screening phase of the study, providing an overview of baseline demographic characteristics and the prevalence of vitamin D deficiency and insufficiency among people screened for the trial.

## Methods

The trial strata were older adults (65 years and above) and ethnic adults aged 18 years and above with a darker skin pigmentation, Fitzpatrick classes IV, V, VI. The inclusion criteria encompassed individuals currently residing in Britain, including those aged 65 years or older of any ethnic background, as well as individuals aged 18 years or older with mixed, multiple, or minority ethnic heritage. All participants were screened for their vitamin D status and only those who had a suboptimal vitamin D status (25(OH)D < 50 nmol/L) were enroled onto the main trial.

Participants were recruited using a multimodal strategy combining digital, community based and in-person approaches. Eligible participants attended an appointment for anthropometric measurements and self-administered finger-prick dried blood spot (DBS) test to assess baseline 25(OH)D levels. Circular holepunches were excised from each DBS card then extracted with water, internal standard (D6-25-hydroxyvitamin D3), zinc sulphate, and methanol, followed by hexane extraction. Extracts were derivatized with PTAD, dried under nitrogen, and reconstituted in 7:3 acetonitrile: water prior to analysis.

Quantitation of 25(OH)D_2_ and 25(OH)D_3_ was performed by liquid chromatography tandem mass spectrometry (LC-MS/MS) and combined to express the total 25(OH)D (nmol/L). Samples were analysed alongside triplicate quality controls and a six-point calibration curve (1/x weighting) on an Agilent 1100 HPLC coupled to a Sciex API-4000 mass spectrometer. Standards and controls were sourced from Chromsystems Instruments & Chemicals GmbH (Am Haag 12, 82166 Gräfelfing/Munich). All samples were analysed independently by SureScreen Scientifics Ltd (Morley Retreat, Church Lane, Morley, Derbyshire, DE7 6DE) using a validated analytical protocol [9]. Only 0.67% of samples were below the method’s limit of detection; null values were retained within the dataset for statistical analysis, and no observations were excluded.

The study was approved by Newcastle University Faculty of Medical Sciences research ethics committee, in line with the Declaration of Helsinki, reference: 2922/50103.

## Results

**Fig. 1:**
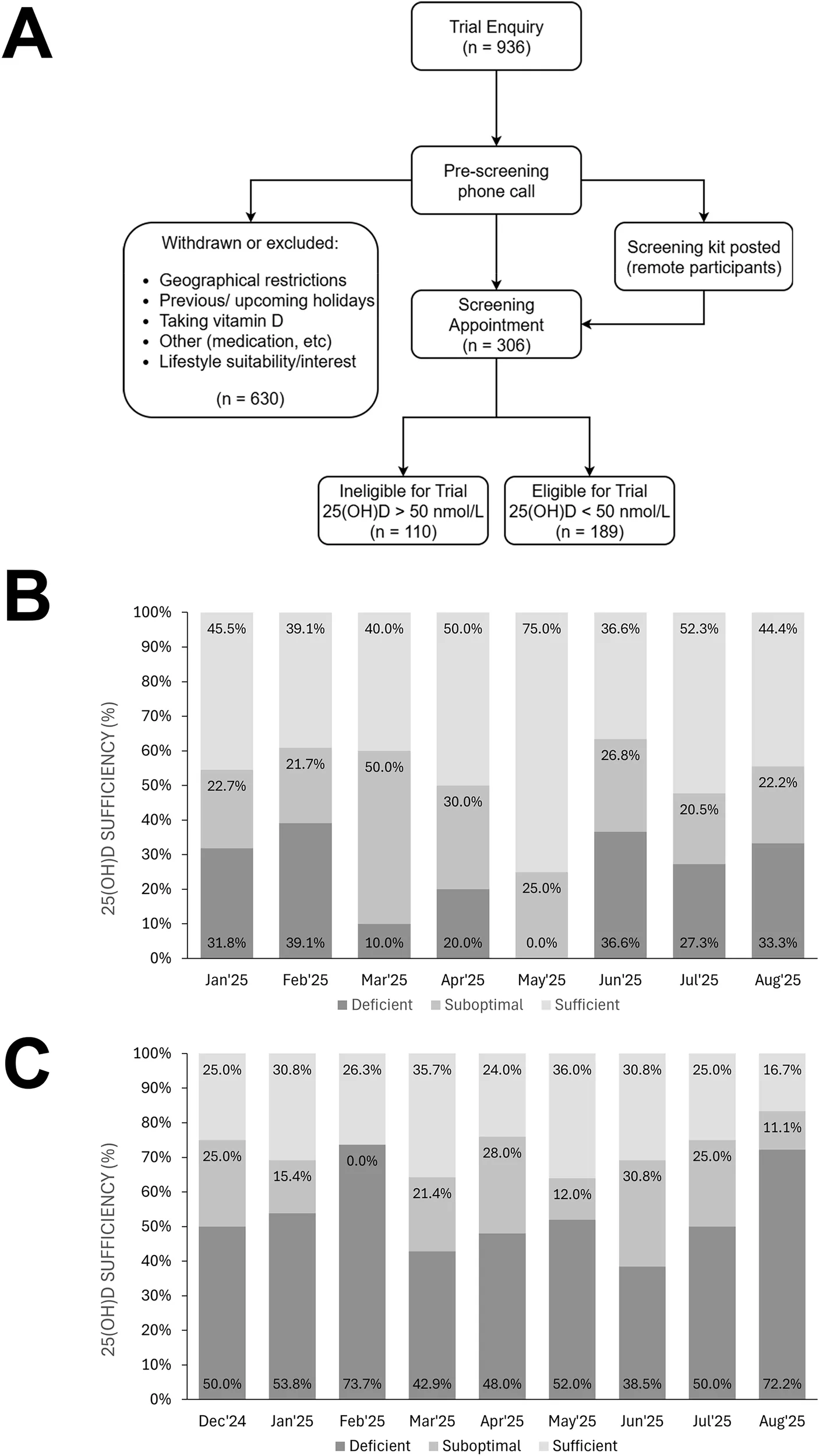
Summary of key study outcomes. Panel **A** shows the CONSORT for participants included in this study. Panel **B** shows the prevalence of vitamin D insuficiency and deficiency by month of recruitment in the older adult stratum of the of the study. Panel **C** shows the prevalence of vitamin D insuficiency and deficiency by month of recruitment in the ethnic adults stratum of the of the study.

Recruitment opened in December 2024 and closed in August 2025. In total, 936 candidates approached the study, with 630 candidates being excluded due to ineligibility. The most common exclusion criteria were already taking cholecalciferol supplementation and withdrawal due to disinterest or lifestyle suitability. In total 299 individuals were screened and their data were included in this analysis. The CONSORT is shown in Fig. 1A. Participant demographics are shown in Table 1. Whilst not a formal exclusion criterion, all older adults enroled in the trial were free living (i.e. not in care homes or sheltered accommodation).

**Table 1:** Summary of demographic profile of the study population.

A			
	Strata I, Adults ≥ 65 Years Old	Strata II, Ethnic Adults ≥ 18 Years Old	*P* value
**n**	168	147	
**Age in years**	69.8 ± 3.75	37.0 ± 14.9	*p* = *0.0000* (t = 26.0, df = 162.27)
**Male**	(*n* = 65) 38.7%	(*n* = 53) 36.1%	*p* = *0.7147* (X² = 0.13363, df = 1)
**Female**	(*n* = 103) 61.3%	(*n* = 94) 63.9%	
**BMI**	28.3 ± 5.49	26.6 ± 5.16	*p* = *0.0050* (t = 2.83, df = 308.63)
**25(OH)D nmol/ L**	49.7 ± 25.9	40.3 ± 29.1	*p* = *0.0028* *(t* = *3.01, df* = *294.46)*
**Mean Difference 25(OH)D nmol/ L**	-9.41 (95% CI -15.6 - -3.26)		
**Asian**	5.35%	64.2%	
**Black**	4.16%	17.0%	
**Caucasian**	89.9%	6.80%	
**Mixed/multiple**	0.60%	10.2%	
**Other ethnicity**	0.00%	1.36%	

B			
	Insufficient 25(OH)D (< 50 nmol /L)	Sufficient 25(OH)D (≥ 50 nmol /L)	*P* value
**n**	189	110	
**Age in years**	50.4 ± 20.3	59.6 ± 17.1	*p* = *0.0060* (t = 2.83, df = 70.00)
**Male**	(*n* = 72) 38.1%	(n = 41) 37.3%	*p* = *0.9858* (X² = 0.000316, df = 1)
**Female**	(*n* = 117) 61.9%	(*n* = 69) 62.7%	
**BMI**	27.2 ± 5.50	27.9 ± 5.36	*p* = *0.8783* (t = 0.154, df = 62.23)
**25(OH)D nmol/ L**	28.7 ± 11.6	73.2 ± 24.2	*p* = *0.0000* (t = –18.1, df = 138.66)
**Mean Difference 25(OH)D nmol/ L**	44.5 (95% CI –49.3 to –39.6)		
**Asian**	37.0%	22.7%	
**Black**	8.99%	7.20%	
**Caucasian**	49.7%	64.5%	
**Mixed/multiple**	3.70%	2.72%	
**Other ethnicity**	5.82%	2.72%	

Continuous variables were assessed using Welch’s *t* test; dichtomised variable was assessed by Pearson’s chi squared test with Yates’ continuity correction.

Prevalence of low 25(OH)D status was assessed month by both across screening dates (December-August 2025). Among all older adults assessed ( ≥ 65 years, *n* = 168), 54.8% were classified as insufficient 25(OH)D (low or deficient) there was little variation by trial reporting month (Fig. 1B). In contrast, ethnic individuals ( ≥ 18 years, *n* = 147), showed consistently higher rates of 25(OH)D insufficiency, with 72.1% classified as low or deficient with no changes in the proportion of individuals who were insufficient across seasons, winter, spring, and summer (Fig. 1C). Recruitment targets were met in August, so no additional data was collected thereon. The prevalence of vitamin D insufficiency within each trial strata, and further subanalyses by ethnic subgroup can be found in Supplementary Fig. S1.

## Discussion

Our study reveals a consistently high and potentially prolonged prevalence of vitamin D insufficiency among Britain’s older and ethnic individuals with monthly 25(OH)D deficiency prevalence rates not falling below 25.0% for older adults and 64.0% for ethnic minorities. Despite the assumption that summer sunlight improves vitamin D status, our data shows that older adults continued to experience relatively high deficiency rates even during summer with an average insufficiency of 55.6% (June-August). This substantially exceeds the approximate 20% year-round deficiency rate observed in the general population [10]. The authors acknowlege that some geographies and disciplines work to different insuffiiciency thresholds, our freely available data support the interpretation of outcomes by peers or groups using other metrics.

More than half of older adults remained deficient even in periods of peak sunlight, indicating that seasonal variation may have limited impact in mitigating insufficiency. The trend was even more pronounced among ethnic individuals. In this strata, deficiency is likely attributed to broader environmental and or lifestyle factors, such as diet, skin pigmentation, and cultural practices [11]. Limitations of this study include lack of data collection on liver function or polypharmacy. No relationship was found between BMI and vitamin D status in exploratory analyses (data not shown).

These findings challenge the reliance on sunlight exposure as the primary strategy for repleting suboptimal 25(OH)D status during summer months in northern Britain. As such, current guidelines on seasonal adjustments to vitamin D intake may not be appropriate for these populations, especially in northern latitudes. It is crucial to consider year-round supplementation and implement more targeted public health strategies [12]. Interventions should address specific dietary needs and cultural barriers, promoting a more comprehensive approach to tackling vitamin D insufficiency across high-risk groups.

## Supplementary information

SUPPLEMENTARY ANALYSES

## Data Availability

Corfe, Bernard (2025). Dataset for screening phase of a clinical trial ISRCTN13778806. figshare. Dataset. 10.6084/m9.figshare.30636023.v1.
