# Correction: Circannual prevalence of vitamin D insufficiency in older and minoritized ethnic adults in Northern Britain: screening outcomes from a clinical trial

**DOI:** 10.1038/s41430-026-01813-3

**Published:** 2026-08-17

**Authors:** Alice Goddard, Anthony Watson, Rowena Tilbury, Bernard M. Corfe, Andrea Fairley

**Affiliations:** 1 Human Nutrition and Exercise Research Centre and School of Biomedical, Nutrition, Sports and Exercise Sciences, Faculty of Medical Sciences, Newcastle University, Newcastle upon Tyne, UK; 2 Human Nutrition and Exercise Research Centre and Population Health Sciences Institute, Faculty of Medical Sciences, Newcastle University, Newcastle upon Tyne, UK

**Keywords:** Randomized controlled trials, Nutrition

Correction to: *European Journal of Clinical Nutrition* https://doi.org/10.1038/s41430-026-01760-z, published online 19 May 2026

In this article, the title was incorrectly given as ‘Circannual prevalence of vitamin D insufficiency in older and minoritized ethnic adults in Northern Britain: screening outcomes from a clinical trial (ISRCTN13778806)’ but should have been ‘Circannual prevalence of vitamin D insufficiency in older and minoritized ethnic adults in Northern Britain: screening outcomes from a clinical trial’.

The original article has been corrected.

